# Innovative Teaching Approaches to Integrating Substance Use Disorder Training into Midwifery and Women's Health Nurse Practitioner Education Programs

**DOI:** 10.1111/jmwh.70038

**Published:** 2025-10-29

**Authors:** Kirby Adlam, Erin Farah, Patrick Thornton, Kelly Rosenberger, Kristen Hufford‐Tims, Pam Pearson, Gina Juliano, Melissa Acuna, Summer Hinthorne, Kylea Liese

**Affiliations:** ^1^ Department of Human Development Nursing Science College of Nursing University of Illinois Chicago Chicago Illinois

**Keywords:** education, midwifery, substance use disorder

## Abstract

Substance use disorder (SUD) is the most common cause of pregnancy‐associated deaths for women in Illinois. In this article, we describe how to provide multimodality education and teaching opportunities in SUD and medication for opioid use disorder, incorporate community outreach to offer new experiential clinical learning opportunities, and enhance preceptor relationships to bolster educational partnerships as an innovative approach to incorporating SUD education into midwifery programs. Our teaching efforts identify the most vulnerable communities and create an innovative, midwifery‐led solution to provide much‐needed culturally aligned care addressing health disparities of those in rural and underserved communities. We highlight our utilization of an online education and training platform that students enroll in to obtain foundational knowledge in medication management for opioid use disorder, describe how we expanded clinical partnerships with providers actively providing medication management for opioid use disorders, and discuss how we evaluated those experiences and provided teaching/learning opportunities for students to present material across multiple cohorts of students. This innovative approach to integrating education for medication management of SUD through the above teaching modalities highlights our ability to meet the needs of our patients and prepare the future midwifery workforce with the clinical skills necessary for the work needed in clinical settings across the United States.

## INTRODUCTION

Substance use disorder (SUD) is the leading cause of pregnancy‐related death, accounting for 32% of all maternal fatalities in Illinois.^1^ This is consistent with nationwide rises in perinatal mortality related to SUD, which rose among women aged 10 to 44 years and more than tripled among perinatal women aged 35 to 44 years.^2^ One in 20 births (5.4%) in Illinois is complicated by SUD.^1^ In rural Illinois, the rate was 12.2%, more than double the state average.^1^ The *Diagnostic and Statistical Manual of Mental Disorders, Fifth Edition* defines SUD as “a cluster of cognitive, behavioral, and physiological symptoms indicating that the individual continues using the substance despite significant substance‐related problems,”^3^ ^(p 484)^ which includes any 10 separate classes of substances, including alcohol and illicit substances.^3^ Opioids are the most common substances related to perinatal mortality in Illinois.^1^ Opioid use during pregnancy is associated with preterm birth, parental overdose, increased rates of neonatal abstinence syndrome, and fetal growth restriction.^4^, ^5^, ^6^ Given the alarming increase in SUD‐related death and sequelae due to opioid use, patients must have access to highly trained and skilled providers during the perinatal period and throughout the life span.

Pregnant and postpartum women are priority populations for SUD treatment services.^7^ The American College of Obstetrics and Gynecology (ACOG) emphasizes that screening with validated tools and medical treatment for SUD should be the standard of care in all pregnancies.^4^ Yet according to the 2022 National Survey on Drug Use and Health, only 15.9% of girls and women over the age of 12 with SUD received treatment.^8^ SUD spans across all racial, socioeconomic, and ethnic groups.^4^ However, Black patients and those from rural populations have higher mortality and morbidity due to SUD, a reflection of systemic barriers to accessing high‐quality care.^1^

  

Continuing education (CE) is available for this article. To obtain CE online, please visit http://www.jmwhce.org. A CE form that includes the test questions is available in the print edition of this issue.

Treatment for SUD improves pregnancy outcomes for women and newborns. The best outcomes are achieved when women are enrolled in comprehensive treatment programs that address physical addiction concurrently with underlying mental health conditions.^2^, ^4^ Prescription opioid agonist therapy prevents withdrawal symptoms, increases prenatal care uptake, and can reduce risks of relapse. Additionally, because pregnant patients with SUD often have comorbid mental health conditions such as depression, anxiety, posttraumatic stress disorder, or a history of trauma, it is imperative to perform comprehensive mental health screenings and refer women to appropriate mental health services.^4^ Education and training in opioid agonist pharmacotherapy during pregnancy and in the postpartum period should be standard in midwifery programs across the nation.^4^

**QUICK POINTS**

✦ Educational institutions can incorporate integrated, experiential substance use disorder (SUD) training into their curricula to enhance midwifery‐led SUD care.
✦ Timely detection and treatment of SUD in the perinatal population impact maternal mortality and morbidity and enhance the health and well‐being of this vulnerable and high‐risk population.
✦ By equipping certified nurse‐midwife and women's health nurse practitioner students with the knowledge and skills to address SUD and medications for opioid use disorder (MOUD), we are preparing them to become health care providers who contribute to reducing maternal morbidity and mortality in their communities.
✦ Advanced practice maternal health care providers are ideally positioned to provide SUD and MOUD care services for women in and beyond the perinatal period.

### Barriers to Care

Disparities in access to comprehensive treatment, including pharmacotherapy for opioid dependence related to social determinants of health, such as race, geography, income, and insurance status, are well documented.^9^ Multifactorial barriers to care have been identified that prevent the comprehensive care necessary to decrease this growing epidemic, but provider education and training in prescription opioid agonist pharmacotherapy should not be one of them.

Lack of confidence and training by health care providers is a significant barrier to comprehensive, quality care. A recent survey of obstetricians and gynecologists found that only 37% were confident in treating opioid use disorders.^10^ Pregnant people with SUD are viewed as high‐risk by physicians and midwives, raising liability concerns and resulting in unnecessary delays in treatment initiation. All health care providers should be competent in providing basic care for patients with SUD, but it is especially important for certified nurse‐midwives (CNMs)/certified midwives (CMs) as primary care providers during pregnancy to possess the education and confidence to screen and initiate treatment.^5^ Appropriate pharmacologic care should not be delayed in pregnancy or any setting while navigating a labyrinth of referrals.

Implicit bias and stigma toward people with SUD, especially pregnant people, are widespread, including among health care providers. ACOG emphasizes the need for providers to prioritize screening and treating in a nonpunitive, nonjudgmental manner.^9^ Screening for pregnant people is only effective if performed in a nonjudgmental, equitable way.^5^, ^9^ Mental health awareness has improved mental health screening and reduced stigma; unfortunately, this has not occurred with perinatal SUD.^11^, ^12^ Many pregnant and postpartum patients worry about judgment and fear punitive policies and actions from health care providers.^12^ This leads to nondisclosure and avoidance of prenatal care for fear of adverse psychosocial outcomes such as involvement of child protection services, criminal justice or legal repercussions, and lack of social support.^12^, ^13^ Normalizing and decriminalizing treatment of SUD during the perinatal period by health care professionals would decrease the barriers these patients face.

### Provider Management of SUD

Policy changes are crucial to addressing the SUD epidemic by expanding access to treatment. In 2016, Congress passed the Comprehensive Addiction and Recovery Act (CARA) in response to increases in mortality from SUD and decreases in providers trained to prescribe buprenorphine to treat patients with SUD.^14^, ^15^ CARA allowed nurse practitioners and physician assistants to apply for a waiver to prescribe buprenorphine after a 24‐hour training course. In 2018, the Substance Use Disorder Prevention that Promotes Opioid Recovery and Treatment for Patients and Communities Act extended prescribing of buprenorphine to CNMs/CMs, clinical nurse specialists, and certified nurse anesthetists.^15^ Elimination of the Drug Enforcement Administration (DEA) X‐Waiver in 2023 removed significant bureaucratic barriers for all prescribing providers, including CNMs and other advanced practice registered nurses (APRNs), to provide buprenorphine, without unnecessary delays for consultation or referrals.^16^ The DEA and Substance Abuse and Mental Health Services Administration (SAMHSA) also provide extensive free online training and continuing education unit materials for any provider wishing to gain appropriate training to prescribe these medications.^16^ This means that all providers who have a current DEA license with Schedule III authority and appropriate training no longer need a waiver to prescribe buprenorphine.

Policies expanding CNM/CM care to include treatment of SUD patients improve overall outcomes. Patients are at an increased risk for SUD during their reproductive years, more than at any other time during their lives.^17^ During the reproductive years, patients often seek care for pregnancy or family planning–related concerns from CNMs/CMs, placing them in a critical position to screen for and treat SUD. Because CNMs/CMs provide prenatal care, this ensures pregnant patients with SUD receive treatment as well as better access to prenatal, postpartum, and primary health care. These providers also provide crucial relationships with treatment centers and referrals to physician collaborators if needed.^15^

## IMPLEMENTING AN INNOVATIVE TRAINING PROGRAM

To bridge the knowledge gap for health care providers caring for perinatal patients suffering from SUD, faculty at the University of Illinois at Chicago (UIC) College of Nursing established an innovative educational experience within the accredited CNM and women's health nurse practitioner (WHNP) programs to prepare students in holistic SUD care. Quality screening for SUD is a core competency for midwifery education^18^; however, CNM and WHNP programs lacked a curriculum that gave future providers the confidence and tools to initiate care and provide ongoing support and treatment. This program was designed to improve perinatal SUD outcomes, decrease health disparities, and reduce stigma of SUD treatment.

Our program, Increasing the Maternal Health care Advanced Practice Nursing Workforce (IMPACT), was awarded a grant through the Advanced Nursing Education Workforce Program, HRSA‐23‐014. The intention of the funding was to support CNM and WHNP students, especially those desiring to serve underserved and rural populations, expand the midwifery workforce, and develop and improve clinical and precepting networks. Although this funding has been essential for relieving financial concerns for students by providing substantial stipends, other CNM/CM programs can incorporate these innovative teaching strategies and expand experiential opportunities without any additional monetary support. In fact, our program plans to continue these learning opportunities regardless of future grant funding.

All students in the CNM and WHNP programs were initially assigned a series of self‐directed, online modules that covered a wide range of topics from stigma and substance abuse to the implementation of SUD services.^19^ These modules provided essential knowledge on addiction, medication management, and prescriptive authority, allowing students to review material at their own pace and as often as needed. To assess understanding, discussions and questions were incorporated into management courses. By embedding content within the management curriculum, we ensured a seamless integration of this material while emphasizing its importance as foundational knowledge for all students.

### Integrating New Clinical Sites

Using Kolb's Experiential Learning Theory, the faculty felt that concrete experiences working with patients would enhance a deeper understanding and appreciation of patients with SUD and how to treat them.^20^ This theory provides a foundation for understanding that experiential learning is necessary to integrate abstract concepts into practice.^20^ Collaborative relationships were established with local physicians, CNMs, and other APRN preceptors providing care for patients with SUD and other mental health disorders. These practice sites varied in both patient population and setting: inpatient, outpatient, and a mobile SUD unit. All grant participants were required to participate in experiential clinical learning opportunities. During the first year of the grant program, the requirement for each student was to attend 2 clinical days. During the second year, clinical sites were added, so the requirement increased to 3 clinical days per year. Using a standardized clinical evaluation form, we captured feedback after each completed clinical rotation and module.

Students are encouraged to apply knowledge gained from the online modules by engaging in clinical experiences and group projects, translating theory into practice. For example, they might draw from the modules on medication management when working with patients in the clinical setting, or they may apply their understanding of stigma and cultural competence when interacting with diverse populations. Our curriculum will continue to incorporate the existing content while also covering emerging topics, such as integrating trauma‐informed care, antiracism, and harm reduction strategies related to SUDs.

### Evolution of the Program

As the program evolved, students became more active in synthesizing and sharing knowledge. In year 2 of the program, faculty developed and implement a SUD simulation day. During this 4‐hour event, students partnered to give presentations on content areas of SUD, allowing a deeper dive into specific topics such as “Medications for Opioid Use Disorder” and “Integrating Opioid Use Disorder Treatment into Clinical Care,” collaboration with peers, and showcasing their understanding and experiences. This learning opportunity reinforced the material and encouraged an interactive, team‐based approach, enhancing individual comprehension and collective knowledge within the cohort.

Integrating another innovative learning experience, the faculty partnered with the local Illinois Affiliate of the American College of Nurse‐Midwives to support a large continuing education event, bringing in an expert provider to discuss SUD and treatment in the perinatal population. This opportunity allowed students to ask questions from experts and network with other experienced providers and preceptors in a collegial, professional atmosphere. Students could see how experienced providers expand their practice through continued education and incorporate this material into their practice. This then allowed students to discuss with their own preceptors how and when this might be implemented in their primary clinical experiences.

### Student Reflections

Students’ evaluations of their SUD learning experiences have been consistently positive since the program's implementation. When new clinic sites were established and additional teaching and learning opportunities became available, students were eager to participate. One student stated, “I am more motivated than ever to include caring for patients with SUD in my future practice.”

Seeing the patients in a clinic setting changed some students’ perspectives. As one student reflected,

It was made very clear to me that many providers are not educated and therefore refuse to utilize Suboxone in clinical settings, because it is simply unfamiliar, and they are not comfortable prescribing it. There is a clear need for educated providers to help manage substance use disorders the correct way. After participating in this IMPACT Scholarship program, I am definitely more likely to care for people with SUD, because I have been exposed to the literature and have had experiences that help me to understand the importance of appropriate SUD treatment.

Students also reflected on the bias and stigma they initially had and how this clinical opportunity affected them: “Each encounter assists in helping me be more open‐minded.” Another student stated, “There is a vast difference between learning about SUD from an online module and caring for patients in person. I greatly appreciated my preceptor's neutrality surrounding sobriety, which I believe made her patients feel comfortable and safe in her care.”

Student feedback supports the expansion of innovative midwifery education and is a crucial component to implementing and standardizing SUD curricula.

## BARRIERS AND LIMITATIONS

Despite the program's success and continued growth, several challenges emerged during the integration of SUD content into the existing CNM curriculum. A primary barrier was the limited availability of clinical sites and preceptors, as students compete regionally for experiential learning placements. To address this, partnerships were developed with more geographically distant sites that offered unique opportunities, such as inpatient experiences. Fortunately, students generally accepted the travel demands in exchange for these positive learning opportunities. Additionally, incorporating new SUD content required coordinated faculty effort to ensure cohesive integration.

This program is restricted to WHNP and CNM students, which is another limitation. Until SUD care is fully integrated across health care specialties and subdisciplines, patients will encounter bias and stigma that delays care.

## THE IMPACT OF SUD TREATMENT TRAINING IN MIDWIFERY PROGRAMS

CNMs/CMs are uniquely positioned to identify, manage, and support perinatal patients experiencing SUD. At the front line of our national health care system, these providers serve as primary care providers offering comprehensive health care to women throughout the reproductive life span, including those affected by SUD. Integrating SUD education and medications for opioid use disorder (MOUD) training into CNM/CM education is crucial to expanding access to care, yet many lack the necessary training and confidence to provide evidence‐based treatment. By integrating SUD education, MOUD prescription training, clinical exposure, and education regarding the psychosocial aspects of SUD into educational programs, midwives will be better equipped to initiate treatment and provide ongoing support. Practice writing prescriptions and managing medication therapy during clinical training fosters confidence and competence in addressing SUD within patient care. CNMs/CMs trained in MOUD can prescribe and manage medications such as buprenorphine, an essential component of SUD treatment. By offering integrated services within prenatal, postpartum, and preventive care, we can reduce barriers to treatment and improve maternal and neonatal outcomes nationwide. Additionally, this expansion in education and training positions CNMs/CMs at the forefront of compassionate, effective care for individuals with SUD, ultimately fostering healthier communities.

### Components of SUD Training Program in Midwifery Education

To enhance CNM/CM‐led SUD care, educational institutions must incorporate integrated, experiential SUD training into their curricula. The faculty at UIC College of Nursing believe that one structured path others can follow to achieve this is through the following: (1) didactic coursework: courses on addiction medicine, behavioral health, and pharmacotherapy should be standard in midwifery programs; (2) clinical exposure: students should have rotations in settings where they can observe and participate in SUD treatment using MOUD; (3) interprofessional collaboration: training should include opportunities to work alongside addiction specialists, social workers, and mental health professionals to reinforce a team‐based approach to care; and (4) continuing education: encouraging CNMs/CMs to obtain additional credentials in addiction medicine and MOUD prescribing enhances their confidence and ability to deliver comprehensive care.

## CONCLUSION

Our efforts to provide multimodality education and teaching opportunities, community building to provide new experiential clinical learning opportunities, and enhancing preceptor relationships to bolster educational partnerships have enabled us to have an innovative approach to teaching SUD education for new health care providers. We created an innovative solution to provide much‐needed, culturally aligned care addressing health disparities in rural and underserved communities.

The IMPACT Program aims to improve the timely detection of SUD and address maternal mortality and morbidity, including neonatal abstinence syndrome, while connecting those identified with emergent health care needs to appropriate resources and support, enhancing the health and well‐being of this vulnerable and high‐risk population. By equipping students with the knowledge and skills to address SUD and its impact on maternal health, we are preparing them to become effective health care providers and contributing to broader efforts to reduce SUD rates and maternal morbidity and mortality. CNMs/CMs and WHNPs are ideally positioned to provide care for patients experiencing SUD in and beyond the childbearing cycle.

Our innovative approach to implementing this curriculum can serve as a blueprint for other accredited midwifery institutions. Given SUD is a national maternal health problem, more providers must be exposed to this type of experiential, comprehensive learning to expand mental health screening and SUD treatment initiation and bring these services to vulnerable patients when and where they are needed most.

## CONFLICTS OF INTEREST

The Health Resources and Services Administration (HRSA), Department of Health and Human Services (HHS) provided financial support for this program. The contents of this article are those of the authors. They may not reflect the policies of HRSA, HHS, or the US Government. The authors have no other disclosures or conflicts of interest.
